# Study of purinosome assembly in cell-based model systems with *de novo* purine synthesis and salvage pathway deficiencies

**DOI:** 10.1371/journal.pone.0201432

**Published:** 2018-07-30

**Authors:** Veronika Baresova, Vaclava Skopova, Olga Souckova, Matyas Krijt, Stanislav Kmoch, Marie Zikanova

**Affiliations:** Research Unit for Rare Diseases, Department of Paediatrics and Adolescent Medicine, First Faculty of Medicine, Charles University and General University Hospital, Prague, Czech Republic; University of Nebraska Medical Center, UNITED STATES

## Abstract

**Background:**

The enzymes involved in *de novo* purine synthesis (DNPS), one of the basic processes in eukaryotic cells, transiently and reversibly form a dynamic multienzyme complex called the purinosome in the cytoplasm. The purinosome has been observed in a broad spectrum of cells, but some studies claim that it is an artefact of the constructs used for visualization or stress granules resulting from the exposure of cells to nutrient-reduced growth media. Both may be true depending on the method of observation. To clarify this point, we combined two previously used methods, transfection and immunofluorescence, to detect purinosomes in purinosome-free cells deficient in particular DNPS steps (CR-DNPS cells) and in cells deficient in the salvage pathway, which resulted in construction of the purinosome regardless of purine level (CR-HGPRT cells).

**Methods and findings:**

To restore or disrupt purinosome formation, we transiently transfected CR-DNPS and CR-HGPRT cells with vectors encoding BFP-labelled wild-type (wt) proteins and observed the normalization of purinosome formation. The cells also ceased to accumulate the substrate(s) of the defective enzyme. The CR-DNPS cell line transfected with a DNA plasmid encoding an enzyme with zero activity served as a negative control for purinosome formation. No purinosome formation was observed in these cells regardless of the purine level in the growth medium.

**Conclusion:**

In conclusion, both methods are useful for the detection of purinosomes in HeLa cells. Moreover, the cell-based models prepared represent a unique system for the study of purinosome assembly with deficiencies in DNPS or in the salvage pathway as well as for the study of purinosome formation under the action of DNPS inhibitors. This approach is a promising step toward the treatment of purine disorders and can also provide targets for anticancer therapy.

## Introduction

**Fig 1:**
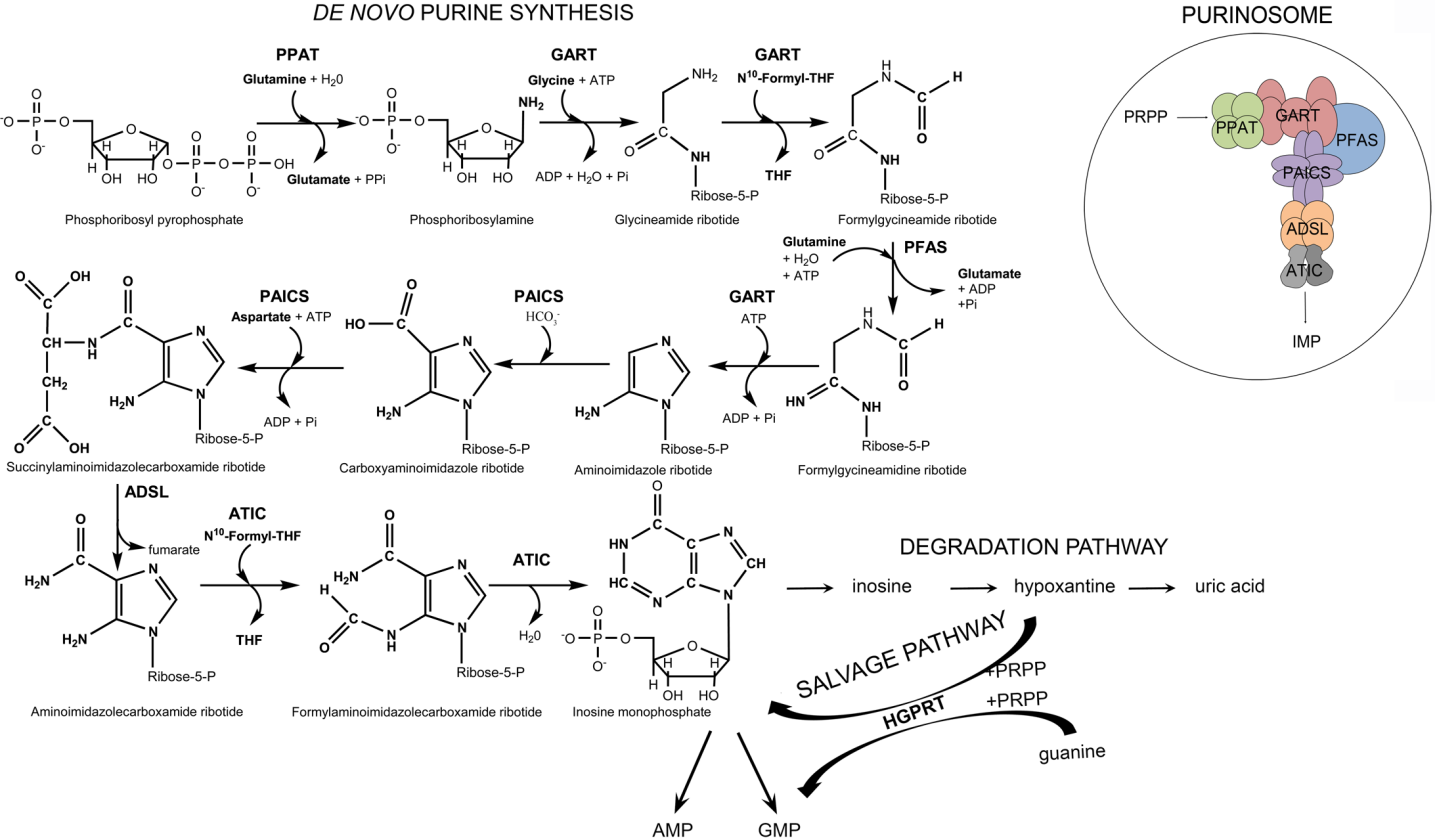
Scheme of *de novo* purine synthesis (DNPS), the salvage pathway, the degradation pathway and the purinosome. The initial substrate in DNPS is phosphoribosyl pyrophosphate (PRPP). Six enzymes are involved in DNPS and the purinosome multienzyme complex: phosphoribosyl pyrophosphate amidotransferase (PPAT), the trifunctional enzyme GART (glycinamide ribonucleotide synthetase/glycinamide ribonucleotide transformylase/aminoimidazole ribonucleotide synthetase), phosphoribosylformylglycinamidine synthetase (PFAS), the bifunctional enzyme PAICS (phosphoribosylaminoimidazole carboxylase/phosphoribosylaminoimidazolesuccinocarboxamide synthetase), adenylosuccinate lyase (ADSL), and the bifunctional enzyme ATIC (5-aminoimidazole-4-carboxamide ribonucleotide transformylase/inosine monophosphate cyclohydrolase). The final product is inosine monophosphate (IMP). IMP is converted into adenosine monophosphate (AMP) and guanosine monophosphate (GMP) and is also degraded to uric acid via the degradation pathway. The hypoxanthine intermediate can be recycled by the enzyme hypoxanthine-guanine phosphoribosyltransferase (HGPRT) into IMP or GMP.

Purines, essential molecules for the synthesis of nucleic acids, universal carriers of chemical energy and components of signalling molecules in all living organisms, are synthesized in higher eukaryotes via 10 reaction steps catalysed by six enzymes, four of which are multifunctional. Once synthesized, they are efficiently recycled by the enzymes of the salvage pathway and eventually removed from cells in the form of uric acid or allantoin (Fig 1).

An important conceptual question is whether the purine-synthesizing enzymes are organized and interact directly within the cell. Because *de novo* purine synthesis (DNPS) produces unstable and/or toxic intermediates [1], the enzymes would need proximity to ensure this vital metabolic function. Knowledge of the composition and regulation of this multienzyme structure, the purinosome, would have important implications regarding human diseases and the treatment of cancer, inflammation and infections. The existence of purinosome has been therefore addressed by various biochemical, molecular and structural approaches [2].

The first direct evidence of purinosome formation was the detection of the spatial signal overlap of transiently expressed fluorescently labelled DNPS proteins in HeLa cells grown in purine-depleted media [3]. This model and its eventual utility for further research on purinosome structure and regulation has however been challenged. The formation of the purinosome bodies has been attributed to the aggregation of overexpressed proteins and to stress granules resulting from the exposure of cells to dialyzed and therefore nutrient-depleted growth media [4].

Further studies with transiently expressed fluorescently labelled DNPS proteins showed that a microtubule network appears to physically control the spatial distribution of purinosomes in the cytoplasm [5], that purinosomes colocalize and can be isolated together with mitochondria [6] and that they differ in size and cell density from stress granules and aggresomes [7].

Another way to detect purinosome complexes is the immunofluorescent labelling of endogenous proteins involved in the DNPS pathway. By this method, purinosome formation was observed in several cell types, including both cancer cell lines and primary cells, grown in purine-depleted medium [8]. The detection of endogenous proteins avoided the need for artificial protein overexpression. The disadvantage of this method is the inability to study purinosome formation *in vivo*.

Recently, we have developed model HeLa cell lines with DNPS enzyme knockouts. These cells represent human purinosome-free model systems [9]. In this work, we prepared model HeLa cells deficient in hypoxanthine-guanine phosphoribosyltransferase (HGPRT, EC 2.4.2.8), one of the key enzymes in a purine salvage pathway, that form purinosomes regardless of the level of purines in the medium. We combined both methods previously used for purinosome study, transfection and immunofluorescence, to investigate the effect of transiently expressed, fluorescently labelled recombinant wild-type (wt) and mutant human DNPS proteins on the formation of purinosome bodies in cells that otherwise never or always formed purinosomes.

## Materials and methods

dx.doi.org/10.17504/protocols.io.qxfdxjn

### Chemicals

Dulbecco's minimum essential medium (DMEM), F12 nutrition mix and foetal bovine serum (FBS) were obtained from Thermo Fisher Scientific, MA, US. All other chemicals were purchased from Sigma-Aldrich, Czech Republic.

### HeLa cell line

The General University Hospital in Prague and Charles University, First Faculty of Medicine has possessed the HeLa cell line for many years; the original cells were obtained from ATCC (American Type Culture Collection).

### CR-HGPRT cell preparation

We used the GeneArt® CRISPR Nuclease Vector with an OFP Reporter Kit (Life Technologies) to knockout the *HPRT1* gene in HeLa cells as described previously [9]. The sgRNA target sequence was designed by the program http://crispr.mit.edu/. We tested the cells for the presence of mutations, HGPRT enzyme activity and thioguanine inhibition.

### Mutation analysis

We isolated gDNA and total RNA from HeLa cells according to standard procedures and transcribed the mRNA into cDNA with the ProtoScript^®^II Reverse Transcriptase Kit (New England BioLabs, MA, United States). PCR analysis was performed in 25 μl reaction mixtures containing Red PCR Master Mix (Rovalab, Germany), 1.5 mM MgCl_2_, 8% DMSO and 0.4 μM specific primers. We gel-purified the amplification products and sequenced the amplicons by the ABI BigDye method (Thermo Fisher Scientific).

### HGPRT enzyme activity assay

The reaction was performed for 1 h at 37°C in a 40 μl reaction mixture containing 50 mM Tris (pH 7.4), 10 mM MgCl_2_, 1.5 mM phosphoribosyl pyrophosphate (PRPP), 1 mM hypoxanthine, 37.5 mM KH_2_PO_4_ and cell lysate (4 μg of the protein). We measured the concentration of inosine monophosphate (IMP) formed using the previously described HPLC-DAD method [10]. The retention time of IMP was 10.8 min.

### Thioguanine inhibition method and growth curves

First, 5 x 10^4^ normal HeLa or CR-HGPRT cells were seeded in 6-well plates containing normal growth medium or medium supplemented with 0.03 mM 6-thioguanine (TG). Every 24 h, the full amount of cells grown in one well was harvested into 1 ml of growth medium and counted with a LUNA™ Automated Cell Counter (Logos Biosystems, Korea). Growth curves were established from the number of cells in 1 ml.

### Cell culture

CRISPR-edited HeLa cells deficient in the trifunctional enzyme GART (glycinamide ribonucleotide synthetase (EC 6.3.4.13)/glycinamide ribonucleotide transformylase (EC 2.1.2.2.)/aminoimidazole ribonucleotide synthetase (EC 6.3.3.1)), phosphoribosylformylglycinamidine synthetase (PFAS, EC 6.3.5.3), the bifunctional enzyme PAICS (phosphoribosylaminoimidazole carboxylase (EC 4.1.1.21)/phosphoribosylaminoimidazolesuccinocarboxamide synthetase (EC 6.3.2.6)), adenylosuccinate lyase (ADSL, EC 4.3.2.2), the bifunctional enzyme ATIC (5-aminoimidazole-4-carboxamide ribonucleotide transformylase (EC 2.1.2.3)/inosine monophosphate cyclohydrolase (EC 3.5.4.10)), or HGPRT were maintained in DMEM/F12 nutrient mixture supplemented with 10% foetal bovine serum (FBS), 1% penicillin/streptomycin and 0.03 mM adenine. DMEM supplemented with dialyzed 10% FBS and 1% penicillin/streptomycin served as the purine-depleted media. The FBS was dialyzed against 0.9% NaCl at 4°C for 48 h with a 10 kDa MWCO dialysis membrane to remove purines.

### Cloning of mammalian expression plasmids

Wild-type (wt) DNPS genes were amplified from previously prepared pMAL-c2wt vectors [9, 11] and inserted into the pTagBFP-C vector (Evrogen, Russia). The vector pTagBFP_Y114H_ADSL was prepared by introducing the mutation *c340T>C* (p.Y114H) into the initial vector pTagBFP_ADSL by using the GeneArt Site-Directed Mutagenesis System (Thermo Fisher Scientific) according to standard procedures. All sequences were verified by DNA sequencing.

### Transfection

For transfection, 1 x 10^5^ CRISPR-edited HeLa cells were transiently transfected with 1.5 μg of constructs using the Neon^®^ Transfection System (Thermo Fisher Scientific) with the following parameters: pulse voltage: 1400 V, pulse width: 20 ms, number of pulses: 2, tip type: 10 μl and seeded on 1.7 cm^2^ glass chamber slides (Thermo Fisher Scientific). After 24 h, the cells were washed and subsequently incubated with purine-depleted or purine-rich medium. After another 24 h, immunofluorescence labelling was performed. PAICS-, PFAS-, ADSL- and ATIC- deficient cells transfected with wt proteins were grown on 6-well plates for 24 h in purine-depleted medium and then tested for DNPS substrate accumulation by LC-MS/MS as described previously [9]. The cells were not synchronized for the cell cycle.

### Immunofluorescence

For immunofluorescence labelling, the cells were fixed with 4% paraformaldehyde in PBS, permeabilized in 0.1% TRITON, washed, blocked with 5% BSA in PBS and incubated in a humidified chamber for 1 h at 37°C with the following primary antibodies: rabbit polyclonal IgG anti-PPAT (Sigma-Aldrich), mouse polyclonal IgG anti-GART (Abnova, Taiwan), and mouse monoclonal IgG2κ anti-PAICS (Sigma-Aldrich). For fluorescence detection, species-appropriate Alexa Fluor^®^ 488 and 555 secondary antibodies (Thermo Fisher Scientific) were used. Slides were mounted with ProLong^®^ Gold Antifade Mountant (Thermo Fisher Scientific) as the fluorescence mounting medium and analysed by confocal microscopy. All immunofluorescence experiments were repeated at least twice.

### Image acquisition and analysis

The prepared slides were analysed by confocal microscopy. XYZ images were sampled according to the Nyquist criterion by using a LeicaSP8X laser scanning confocal microscope with an HC PL APO objective (63x, N.A. 1.40) and 405, 488 and 543 laser lines. The images were restored by a classic maximum likelihood restoration algorithm in Huygens Professional Software (SVI, Hilversum, The Netherlands) [12]. Colocalization maps with single-pixel overlap coefficient values ranging from 0–1 [13] were created in Huygens Professional Software. The resulting overlap coefficient values are presented in pseudocolour, and the scale is shown in the corresponding lookup tables (LUT). Image analysis was performed on at least ten cells from each cell type. The conditions for image acquisition were the same for all cells included in the experiment.

## Results

### Preparation of HeLa cells deficient in HGPRT (CR-HGPRT cells)

**Fig 2:**
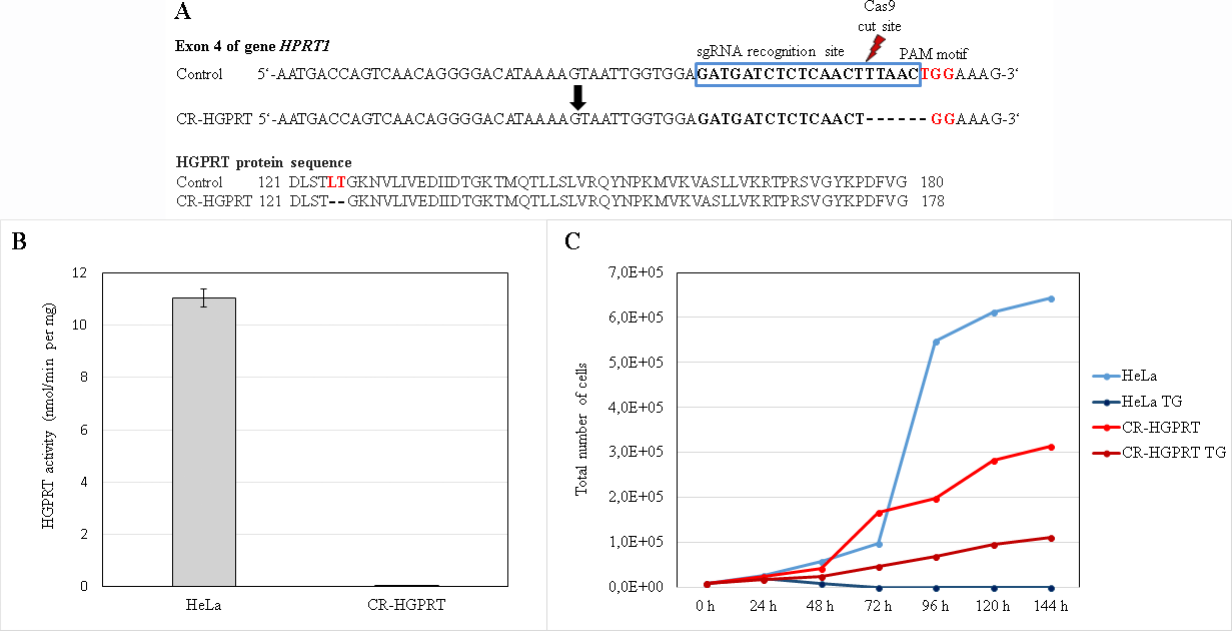
Characterization of the HGPRT knockout cells. (A): Illustration of the sgRNA targeting sequence in exon 4 of the *HPRT1* gene in control and CR-HGPRT cells and the protein sequences of wild-type and mutated HGPRT. The 20-bp target sgRNA sequence is indicated in the blue box, adjacent to the NGG (TGG) PAM motif sequence (red coloured). The probable Cas9 cut site is indicated by the red flash-shaped object. CR-HGPRT is shown with the appropriate c.373-378delTTAACT mutation. In the HGPRT protein sequence, the affected amino acids are coloured red; the CR-HGPRT protein shows a p. 125-126del deletion. (B): The activity of the HGPRT enzyme was determined in the control and CR-HGPRT cells. The activity of the enzyme in the CR-HGPRT cells was 0% of the activity in the control cells (n = 3). (C): Growth curves of CR-HGPRT cells and control HeLa cells. The cells were grown in normal growth medium and in growth medium containing 0.03 mM thioguanine (TG). The CR-HGPRT cells grew in the normal growth medium (light red line) and more slowly in the growth medium containing TG (dark red line). All the control cells in the medium containing TG died within 72 h (dark blue line), while the control cells in the normal medium showed ten times more growth at the same time point (light blue line).

We knocked-out the *HPRT1* gene in HeLa cells using the CRISPR-Cas9 genome editing system. We detected positive clones via cDNA and gDNA sequencing, the thioguanine inhibition method and subsequent growth curves and activity assays (Fig 2). The homozygous mutation in the targeted gene *c*. *373_378delTTAACT* (p. 125-126del) (Fig 2A) resulted in the expression of an HGPRT protein with undetectable enzyme activity (Fig 2B). The lack of HGPRT activity was also tested by establishing growth curves with and without the addition of 0.03 mM TG to the growth media. After the addition of TG, normal HeLa cells underwent cell death within 72 h (Fig 2C, dark blue), while CR-HGPRT cells survived for at least 6 days (Fig 2C, dark red). In normal medium, both cell types grew normally (Fig 2C, light red and blue).

### Purinosome formation in CR-HGPRT cells

**Fig 3:**
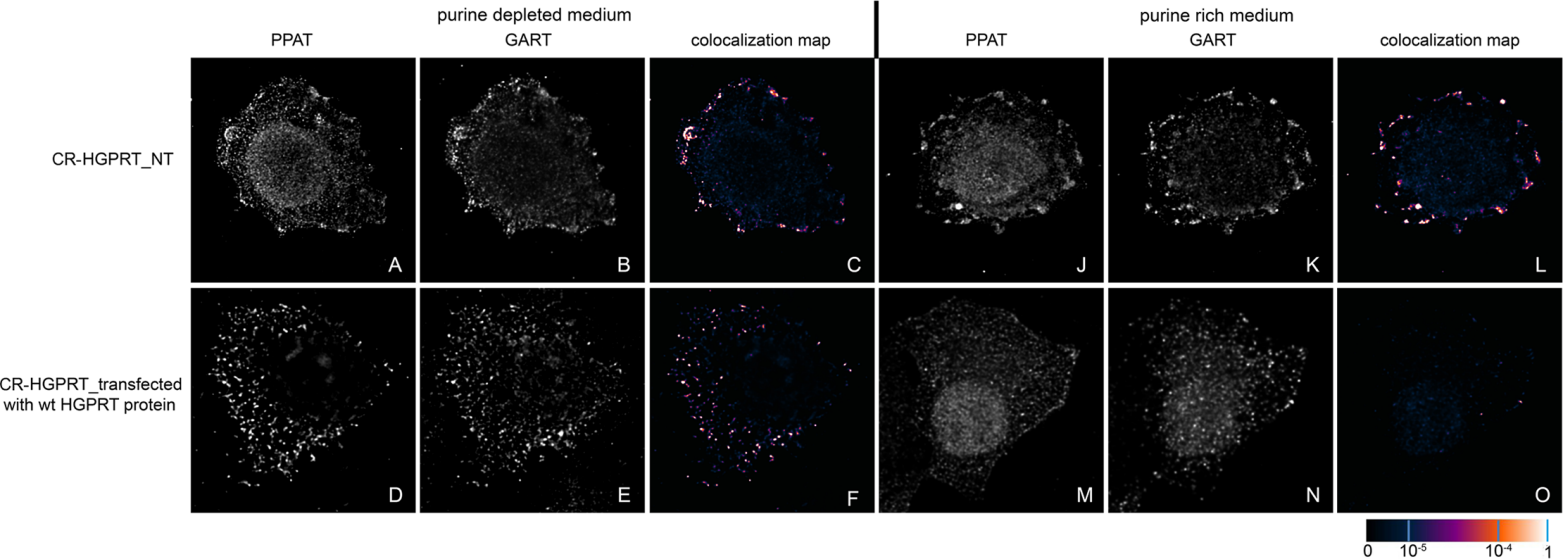
Immunodetection of GART and PPAT in CR-HGPRT cells. Non-transfected CR-HGPRT cells exhibited endogenous GART (B, K) and PPAT (A, J) proteins in the form of fine granules, and their fluorescent signals showed a high degree of overlap in both the purine-depleted medium (C) and the purine-rich medium (L). In cells transfected with a vector encoding the wt HGPRT protein, the endogenous proteins formed fine granules with high signal colocalization in the purine-depleted medium (D, F), whereas in the purine-rich medium, the proteins remained diffuse (M, N) with no colocalization (O). The values of the fluorescent signal overlaps are shown in pseudocolour, and the scale is shown at the lower right in the corresponding LUT.

To determine whether mutations in the *HPRT1* gene affect the intracellular compartmentalization of DNPS proteins *in vivo*, we investigated purinosome formation in CR-HGPRT cells. We cultured HeLa cells for 24 h in purine-rich or purine-depleted media and immunolabelled the combination of the PPAT and GART enzymes. We observed purinosome formation independently of the amount of purines in the growth media (Fig 3A–3C and 3J–3L).

### Transfection of knockout HeLa cells by constructs encoding wt proteins

**Fig 4:**
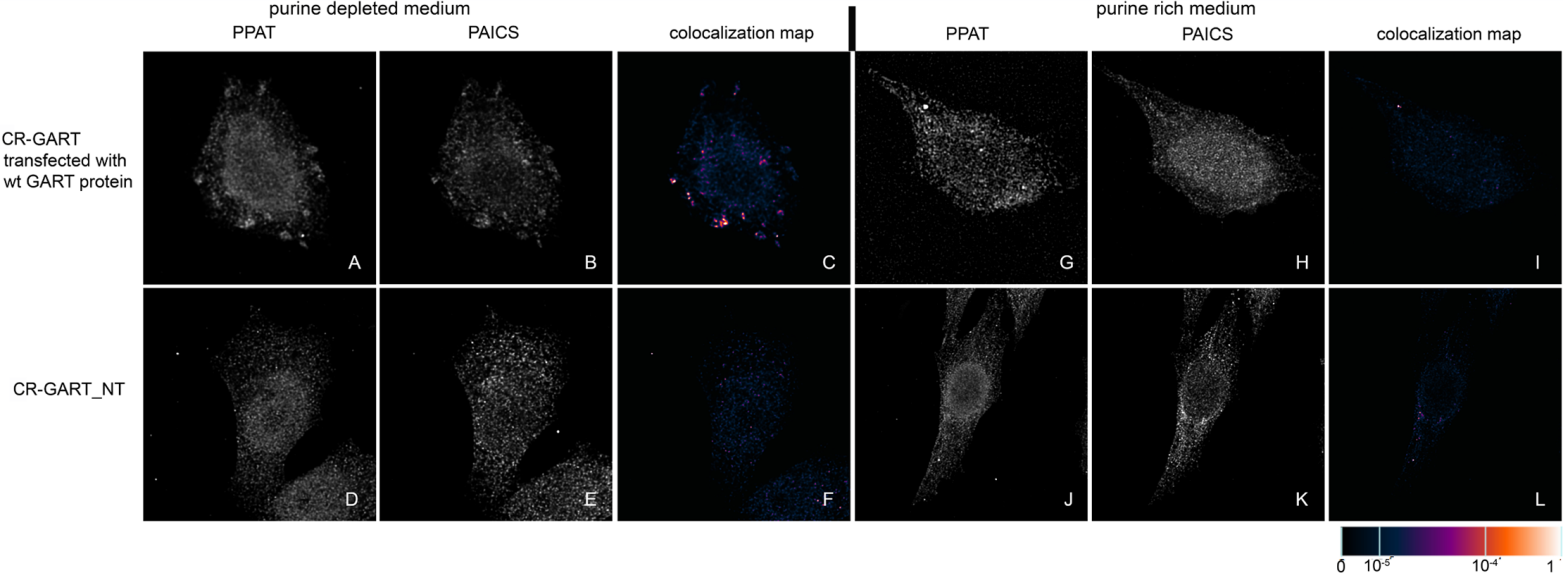
Immunochemical labelling of the endogenous proteins PPAT and PAICS in CR-GART cells transfected with constructs encoding wt-GART proteins. In cells transfected with constructs encoding the wt-GART protein, the endogenous proteins PPAT (A) and PAICS (B) were observed in the form of fine granules with their fluorescent signals showing a high degree of overlap in purine-depleted medium (C), whereas in purine-rich medium, the proteins PPAT (G) and PAICS (H) remained diffuse and did not colocalize (I). The same behaviour was observed in the control HeLa cells (Fig 9). Endogenous proteins in the non-transfected cells remained diffuse regardless of the level of purines in the media (D, E, J, K) and did not colocalize (F, L). The values of the fluorescent signal overlaps are shown in pseudocolour, and the scale is shown at the lower right in the corresponding LUT.

**Fig 5:**
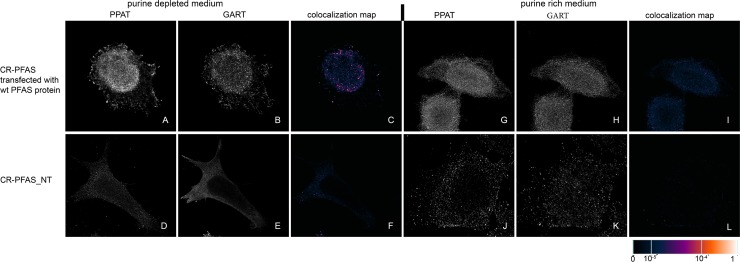
Immunodetection of PPAT and GART in CR-PFAS cells transfected with constructs encoding wt-PFAS proteins. In cells transfected with constructs encoding wt-PFAS protein, the endogenous proteins PPAT (A) and GART (B) were observed in the form of fine granules with their fluorescent signals showing a high degree of overlap in purine-depleted medium (C), whereas in purine-rich medium, the proteins PPAT (G) and GART (H) remained diffuse and did not colocalize (I). The same behaviour was observed in the control HeLa cells (Fig 9). When the cells were not transfected, the endogenous proteins remained diffuse regardless of the level of purines in the media (D, E, J, K) and did not colocalize (F, L). The values of the fluorescent signal overlaps are shown in pseudocolour, and the scale is shown at the lower right in the corresponding LUT.

**Fig 6:**
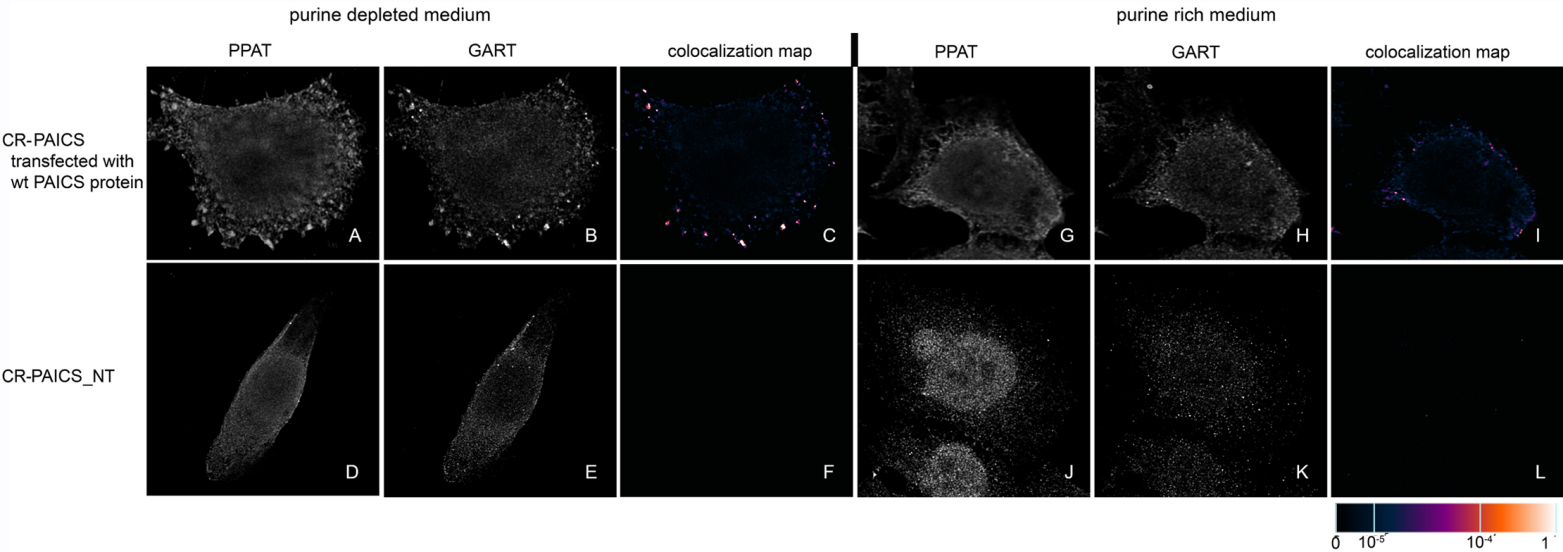
Immunodetection of PPAT and GART in CR-PAICS cells transfected with constructs encoding wt-PAICS proteins. In cells transfected with constructs encoding wt-PAICS protein, the endogenous proteins PPAT (A) and GART (B) were observed in the form of fine granules with their fluorescent signals showing a high degree of overlap in purine-depleted medium (C), whereas in purine-rich medium, the proteins PPAT (G) and GART (H) remained diffuse and did not colocalize (I). The same behaviour was observed in the control HeLa cells (Fig 9). Endogenous proteins in the non-transfected cells remained diffuse regardless of the level of purines in the media (D, E, J, K) and did not colocalize (F, L). The values of the fluorescent signal overlaps are shown in pseudocolour, and the scale is shown at the lower right in the corresponding LUT.

**Fig 7:**
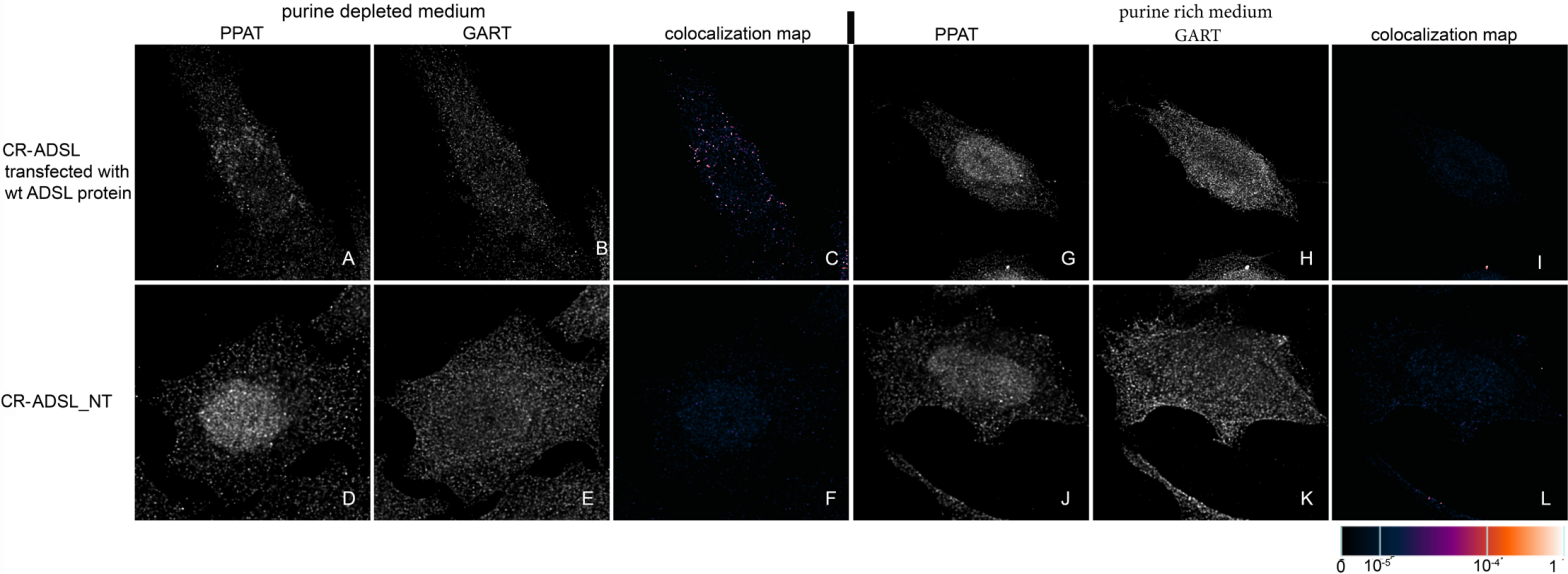
Immunodetection of PPAT and GART in CR-ADSL cells transfected with constructs encoding wt-ADSL proteins. In cells transfected with constructs encoding wt-ADSL protein, the endogenous proteins PPAT (A) and GART (B) were found in the form of fine granules with their fluorescent signals showing a high degree of overlap in purine-depleted medium (C), whereas in purine-rich medium, the proteins PPAT (G) and GART (H) remained diffuse and did not colocalize (I). The same behaviour was observed in the control HeLa cells (Fig 9). When the cells were not transfected, the endogenous proteins remained diffuse regardless of the level of purines in the media (D, E, J, K) and did not colocalize (F, L). The values of the fluorescent signal overlaps are shown in pseudocolour, and the scale is shown at the lower right in the corresponding LUT.

**Fig 8:**
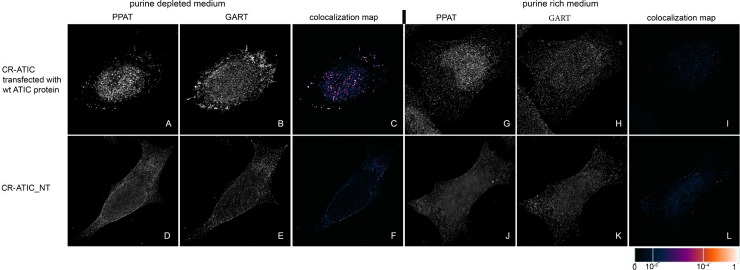
Immunodetection of PPAT and GART in CR-ATIC cells transfected with constructs encoding wt-ATIC proteins. In cells transfected with constructs encoding wt-ATIC protein, the endogenous proteins PPAT (A) and GART (B) were observed as fine granules with their fluorescent signals showing a high degree of overlap in purine-depleted medium (C), whereas in purine-rich medium, the proteins PPAT (G) and GART (H) remained diffuse and did not colocalize (I). The same behaviour was observed in the control HeLa cells (Fig 9). When the cells were not transfected, the endogenous proteins remained diffuse regardless of the level of purines in the media (D, E, J, K) and did not colocalize (F, L). The values of the fluorescent signal overlaps are shown in pseudocolour, and the scale is shown at the lower right in the corresponding LUT.

We transiently transfected previously prepared HeLa cells with the knockout of individual DNPS genes (CR-DNPS cells) [9] and CR-HGPRT cells with vectors encoding pTagBFP-labelled wt proteins to restore or disrupt purinosome formation. We transfected HeLa cells deficient for GART (CR-GART) with the pTagBFP_wt GART vector (Fig 4), HeLa cells deficient for PFAS (CR-PFAS) with the pTagBFP_wt PFAS vector (Fig 5), HeLa cells deficient for PAICS (CR-PAICS) with the pTagBFP_wt PAICS vector (Fig 6), HeLa cells deficient for ADSL (CR-ADSL) with the pTagBFP_wt ADSL vector (Fig 7), HeLa cells deficient for ATIC (CR-ATIC) with the pTagBFP_wt ATIC vector (Fig 8), and CR-HGPRT cells with the pTagBFP_wt HGPRT vector (Fig 3).

**Fig 9:**
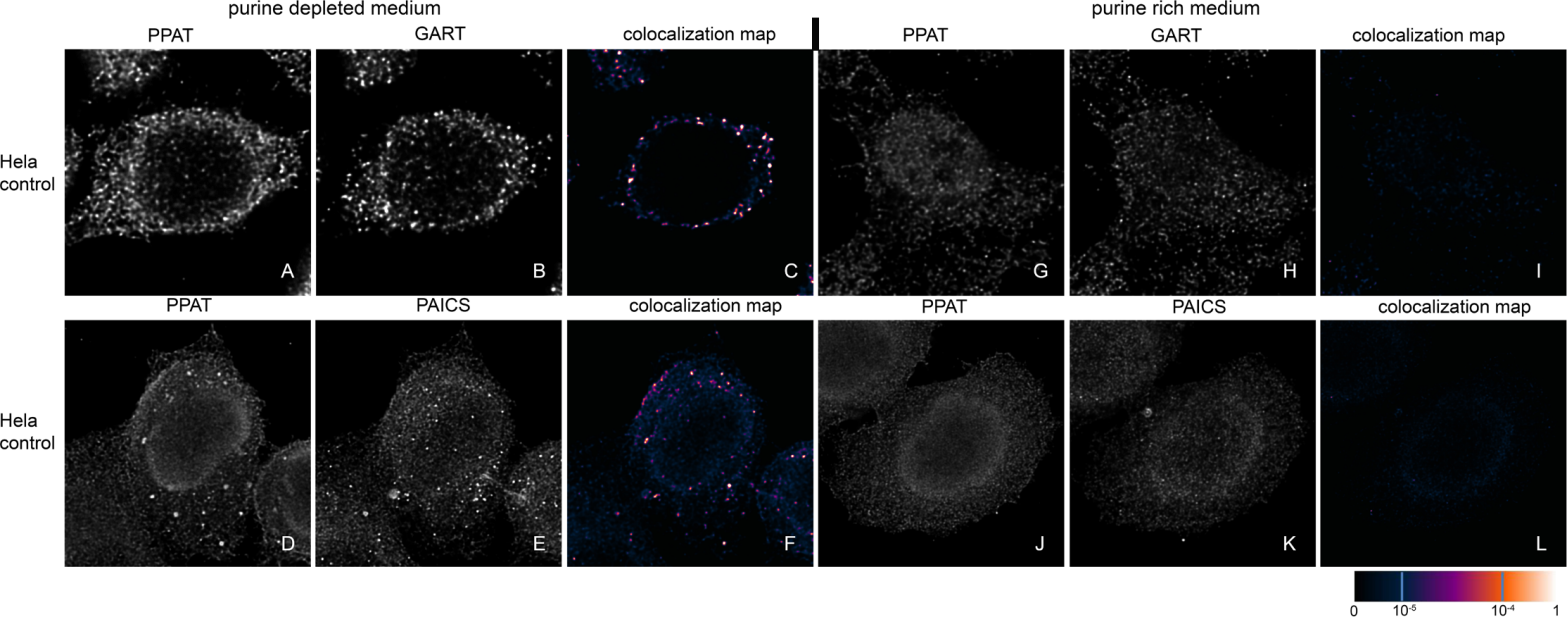
Immunodetection of PPAT, GART and PAICS in control HeLa cells. Control HeLa cells were immunolabelled with PPAT and GART (A, B, C, G, H, I) or with PPAT and PAICS (D, E, F, J, K, L). Both combinations of the endogenous proteins formed granules (A, B, D, E) with high signal colocalization in the purine-depleted medium (C, F), whereas in the purine-rich medium, the proteins remained diffuse (G, H, J, K) with no colocalization (I, L). The values of the fluorescent signal overlaps are shown in pseudocolour, and the scale is shown at the lower right in the corresponding LUT.

All the transfected cells were grown in purine-rich and in purine-depleted medium. After 24 h, we immunofluorescently labelled and observed the colocalization of the DNPS protein phosphoribosyl pyrophosphate amidotransferase (PPAT) with GART (Figs 3 and 5–8) or with PAICS (CR-GART cell line, Fig 4). We observed the restoration of purinosome formation in CR-DNPS cells in the purine-depleted medium (Figs 4–8A–8C), whereas in the purine-rich medium, the proteins remained diffuse (Figs 4–8G–8I). In CR-HGPRT cells transfected with the pTagBFP_wt HGPRT vector, we observed the disruption of purinosome formation in the purine-rich medium (Fig 3M–3O), while purinosome formation remained unchanged in the purine-depleted medium (Fig 3D–3F). The control HeLa cells exhibited similar behaviour to the CR-DNPS and CR-HGPRT cells transfected with wt proteins (Fig 9). We also tested CR- PFAS, CR-PAICS, CR-ADSL and CR-ATIC cells grown in PD media and transfected with wt proteins for DNPS substrate accumulation in the cell lysates. The levels of N-formylglycinamide ribotide/riboside (FGAR/r) decreased after pTagBFP_wt PFAS vector transfection in CR-PFAS. Decreases were also observed in aminoimidazole ribotide/riboside (AIR/r) after pTagBFP_wt PAICS vector transfection in the CR-PAICS cells, succinylaminoimidazolecarboxamide ribotide/riboside (SAICAR/r) and succinyladenosine monophosphate (SAMP)/succinyladenosine (S-Ado) after pTagBFP_wt ADSL vector transfection in CR-ADSL cells and aminoimidazolecarboxamideribotide/riboside (AICAR/r) after pTagBFP_wt ATIC vector transfection in CR-ATIC cells (Table 1, S1 Fig).

**Table 1:** Purine metabolites in cell lysates of non-transfected (non TR) and wt transfected (TR) CR-cells (n = 3).

	HeLa	CR-PFAS		CR-PAICS		CR-ADSL		CR-ATIC	
metabolite	μmol/l	non TR μmol/l	TR μmol/l	non TR μmol/l	TR μmol/l	non TR μmol/l	TR μmol/l	non TR μmol/l	TR μmol/l
FGAr	nd	12.1±2.1	0.32±0.22	0.28±0.06	nd	nd	nd	nd	nd
FGAR	nd	2.1±0.9	0.07±0.06	0.13±0.04	nd	nd	nd	nd	nd
AIr	nd	nd	nd	1.0±0.1	0.004±0.001	nd	nd	nd	nd
AIR	nd	nd	nd	2.93±0.16	0.33±0.01	nd	nd	nd	nd
SAICAr	nd	nd	nd	nd	nd	0.40±0.04	0.072±0.002	0.05±0.01	≈ LOQ
SAICAR	nd	nd	nd	nd	nd	0.21±0.03	nd	nd	nd
S-Ado	≈ LOQ	≈ LOQ	≈ LOQ	≈ LOQ	≈ LOQ	10.5±1.1	1.9±0.2	≈ LOQ	≈ LOQ
SAMP	≈ LOQ	≈ LOQ	≈ LOQ	nd	nd	22.7±2.5	nd	nd	nd
AICAr	nd	nd	nd	nd	nd	nd	nd	0.09±0.03	nd
AICAR	nd	nd	nd	nd	nd	nd	nd	1.2±0.4	nd

nd–not detected

≈ LOQ—Values close to limit of quantification.

### Transfection of ADSL-deficient HeLa cells by a mutant ADSL protein

**Fig 10:**
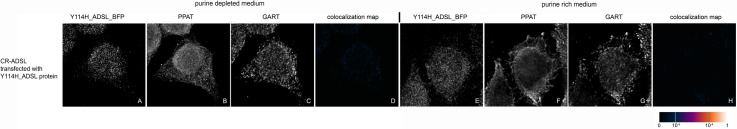
Immunochemical labelling of the endogenous proteins GART and PPAT in CR-ADSL cells transfected with a construct encoding a mutant inactive protein, p.Y114H ADSL. CR-ADSL cells were transfected with a vector encoding the BFP-labelled inactive protein p.Y114H ADSL (A, E) and seeded in purine-depleted medium (A, B, C, D) or purine-rich medium (E, F, G, H). The endogenous proteins PPAT (B, F) and GART (C, G) remained diffuse, and the signals did not colocalize, regardless of the amount of purines in the growth media (D, H). The values of the fluorescent signal overlaps are shown in pseudocolour, and the scale is shown at the lower right in the corresponding LUT.

As a control to examine whether cells undergoing transient transfection and nutrition starvation formed purinosomes or stress bodies, we transfected CR-ADSL cells with the pTagBFP_Y114H_ADSL vector encoding the ADSL enzyme with the mutation Y114H. This mutation is one of the most common mutations in patients suffering ADSL deficiency and results in zero enzyme activity [10]. We used the same conditions as for the transfection of the pTagBFP_wt ADSL vector into the CR-ADSL cells. We observed that the immunofluorescently labelled endogenous proteins remained diffuse regardless of the quantity of purines in the growth media (Fig 10).

## Discussion and conclusions

Purinosomes have previously been detected in cells [3], but the argument was made that they may be artefacts of the constructs used for visualisation [4]. Both conclusions seemed possible depending on the cell state and the method of observation. The aim of this work was to detect purinosome formation by two previously described methods in non-purinosome-forming cells and in cells that form purinosomes regardless of purine levels. Furthermore, we aimed to determine the suitability of these systems for purinosome studies.

We built upon findings that purinosome formation is disrupted in fibroblasts deficient in ADSL and ATIC [8], which are affected by the flow of DNPS intermediates. By contrast, purinosome formation is increased in fibroblasts that are deficient in HGPRT, which results in a nonfunctional salvage pathway and therefore primarily depends on DNPS to generate purine nucleotides [14].

In our previous study, we introduced knockouts of individual DNPS genes into HeLa cells, in which we observed disrupted or greatly reduced purinosome formation (CR-DNPS cells) [9]. In this work, we prepared a CRISPR-Cas9-edited model HeLa cell system in which the purinosome forms independently of the purine level in the growth medium (CR-HGPRT cells).

Using the existing purinosomes-free systems, we checked whether purinosome assembly was restored in CR-DNPS cells grown in purine-depleted medium after transfection with vectors encoding BFP-labelled wt GART, PFAS, PAICS, ADSL or ATIC proteins. We also tested whether purinosome formation was affected in CR-HGPRT cells grown in purine-rich medium after transfection with a vector encoding BFP-labelled wt HGPRT protein.

To demonstrate purinosome formation, we used confocal microscopy and applied an established method of signal colocalization analysis based on single-pixel overlap coefficient values [13]. As the causal genetic defects often affect the amount of the corresponding mutant protein *in vivo*, we studied the intracellular localization of selected combinations of endogenous DNPS enzymes. Using this approach, we detected shared compartmentalization and spatial overlaps that suggested purinosome formation.

Purinosome formation in CR-DNPS cells transfected with constructs encoding wt DNPS proteins was restored to the level in the control HeLa cells in purine-depleted medium, whereas in purine-rich medium, the proteins remained diffuse. To prove that the structural bodies observed were real purinosomes, we transfected ADSL-deficient cells with a vector encoding a mutated ADSL protein (p.Y114H) with low enzyme activity [10]. We did not observe any purinosome assembly, and the proteins remained diffuse. This result is consistent with our knowledge that the fibroblasts of patients with the ADSL mutation Y114H likewise do not form purinosomes [8]. Based on these data, we conclude that purinosome formation by endogenous proteins can be restored by transiently introduced constructs encoding wt proteins. Furthermore, the restoration of purinosome function was verified in CR-DNPS cell lines transfected with wt proteins by measuring the level of substrates normally accumulated by deficient cells [9]. After transfection, substrate accumulation was significantly reduced or not detected.

HGPRT-deficient model cells transfected with a construct encoding the wt HGPRT protein ceased to form purinosomes in a purine-rich medium, which corresponds to the behaviour of healthy HeLa cells. This result supports the theory that the bodies observed are putative purinosomes.

We conclude that both methods, transient transfection and immunofluorescence, are useful for the detection of purinosome formation in HeLa cells. Moreover, the HGPRT knockout model of HeLa cells provides the ability to study purinosome formation in purine-rich medium without the need to expose the cells to stress conditions involving dialyzed and therefore nutrient-depleted growth media.

Cell-based models with specific deficiencies in the DNPS or salvage pathway provide a unique system for evaluating the efficacy and selectivity of DNPS inhibitors and activators at the biochemical level and the purinosome assembly level. The purinosome is a higher level of organization and regulation of metabolic enzymes in purine biosynthesis, and selective DNPS modulators can be used to better understand the role of its formation in both normal and disease states. This approach is a promising step toward the treatment of DNPS disorders and could provide a target for the treatment of cancer in which purine synthesis plays an essential role [15].

## Supporting information

S1 Fig LC-MS/MS chromatograms of purine metabolites in non-transfected and wt transfected CR-cell lines. Metabolites in non-transfected cells are labelled with a dashed line and in transfected cells with a full line. The decrease in FGAR/r levels after pTagBFP_wt PFAS vector transfection in CR-PFAS cell lysate is shown in (A). The decrease in AIR/r levels after pTagBFP_wt PAICS vector transfection in CR-PAICS cell lysate is shown in (B). The decrease in SAICAR/r and SAMP/SAdo levels after pTagBFP_wt ADSL vector transfection in CR-ADSL cell lysate is shown in (C). The decrease in AICAR/r levels after pTagBFP_wt ATIC vector transfection in CR-ATIC cell lysate is demonstrated in (D).
(TIF)
