# Supplementary figures and images for: Study of purinosome assembly in cell-based model systems with de novo purine synthesis and salvage pathway deficiencies
Source: PLoS One. 2018 Jul 30;13(7):e0201432. doi: 10.1371/journal.pone.0201432 (PMC6066232; doi:10.1371/journal.pone.0201432)

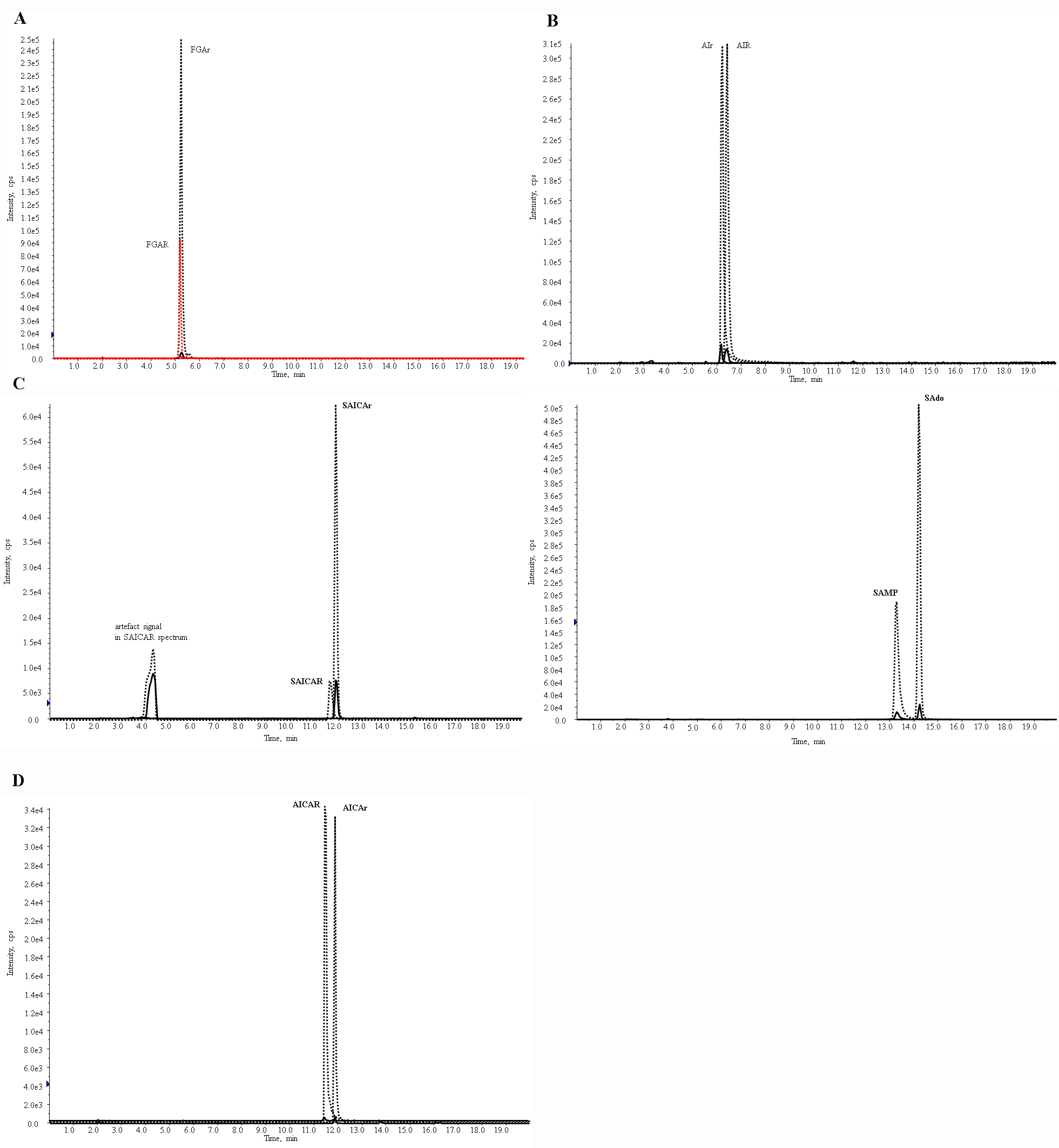

Supplement: S1 Fig — Metabolites in non-transfected cells are labelled with a dashed line and in transfected cells with a full line. The decrease in FGAR/r levels after pTagBFP_wt PFAS vector transfection in CR-PFAS cell lysate is shown in (A). The decrease in AIR/r levels after pTagBFP_wt PAICS vector transfection in CR-PAICS cell lysate is shown in (B). The decrease in SAICAR/r and SAMP/SAdo levels after pTagBFP_wt ADSL vector transfection in CR-ADSL cell lysate is shown in (C). The decrease in AICAR/r levels after pTagBFP_wt ATIC vector transfection in CR-ATIC cell lysate is demonstrated in (D). (TIF) [file pone.0201432.s001.tif]
